# Potential of N_2_ Gas Flushing to Hinder Dairy-Associated Biofilm Formation and Extension

**DOI:** 10.3389/fmicb.2020.01675

**Published:** 2020-07-28

**Authors:** Patricia Munsch-Alatossava, Tapani Alatossava

**Affiliations:** ^1^Independent Researcher, Helsinki, Finland; ^2^Department of Food and Nutrition, University of Helsinki, Helsinki, Finland

**Keywords:** cold chain, food spoilage, dairy, bacteria, biofilms, N_2_ gas flushing

## Abstract

Worldwide, the dairy sector remains of vital importance for food production despite severe environmental constraints. The production and handling conditions of milk, a rich medium, promote inevitably the entrance of microbial contaminants, with notable impact on the quality and safety of raw milk and dairy products. Moreover, the persistence of high concentrations of microorganisms (especially bacteria and bacterial spores) in biofilms (BFs) present on dairy equipment or environments constitutes an additional major source of milk contamination from pre- to post-processing stages: in dairies, BFs represent a major concern regarding the risks of disease outbreaks and are often associated with significant economic losses. One consumption trend toward “raw or low-processed foods” combined with current trends in food production systems, which tend to have more automation and longer processing runs with simultaneously more stringent microbiological requirements, necessitate the implementation of new and obligatory sustainable strategies to respond to new challenges regarding food safety. Here, in light of studies, performed mainly with raw milk, that considered dominant “planktonic” conditions, we reexamine the changes triggered by cold storage alone or combined with nitrogen gas (N_2_) flushing on bacterial populations and discuss how the observed benefits of the treatment could also contribute to limiting BF formation in dairies.

## The Dairy Sector, Between Prospects, and Challenges

The economic importance of the dairy sector is obvious when considering the global milk output estimated at 843 million tons in 2018 (FAO, 2019), or the current projections that predict an increase by 1.7% per annum of the world milk production over the next decade 2019/2028 (OECD-FAO, 2019). The EU, with a production of 172.2 million tons of milk in 2018, represents a major player in the global dairy market as the dairy sector constitutes the second largest in terms of total agricultural output (Augère-Granier, 2018).

Food production systems face growing concerns related to climate change and greenhouse gas emissions, and the dairy sector is also urged to implement sustainable criteria from farms to dairies worldwide, such as to optimize the use of natural resources and reduce the environmental impact (Clay et al., 2020). Another important aspect concerns tremendous food spoilage: globally, one-third of all food produced for human consumption is lost or wasted. In Europe alone, 29 million tons of dairy products are lost or wasted every year; this loss represents a massive squandering of resources (land, water, energy, labor, and capital), including needless production of greenhouse gas emissions that promote global warming and climate change (FAO, 2015). Microbes, by overcoming hurdles applied for food preservation, also significantly contribute to food losses.

## Specific Features of Milk and Its Preservation

**FIGURE 1:**
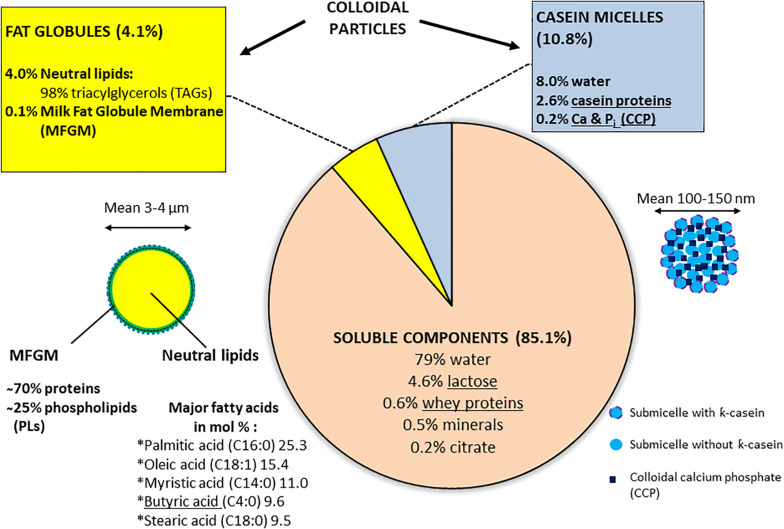
Bovine raw milk composition^a^. ^a^[Singh and Bennett (2002), Tetra Pak (2003), Walstra et al. (2006)]. Some components, which are underlined, support BF formation according to Speers and Gilmour (1985), Barnes et al. (1999), Kim and Lund (2007), Varhimo et al. (2011), Pasvolsky et al. (2014), Flint et al. (2015), Assaf et al. (2015), and Duanis-Assaf et al. (2016).

Milk, which supports the growth of newborn mammals, presents a spectrum and balance of the different components characteristic of each mammalian species and simultaneously carries nutritional components (lipids, proteins, carbohydrates (lactose), vitamins, and salts, etc.), protective functions (linked to the presence of antimicrobial elements), or regulatory functions (growth hormones, regulatory proteins, and bioactive lipids, etc.; Singh and Bennett, 2002; Tetra Pak, 2003; Walstra et al., 2006). The major components of fresh raw milk for bovines, the most important dairy husbandry animals in Europe, are detailed in Figure 1. Milk constitutes an example of a fat-in-water emulsion, where the fat fraction exists mostly as small globules dispersed in the milk plasma. Milk fat is characterized by a variety of more than 400 different fatty acids: among the 5 major types (listed in Figure 1), butyric and oleic acids are in a liquid state at room temperature (Taylor and Mac Gibbon, 2002; Tetra Pak, 2003). The milk fat globules are delimitated by an 8–10-nm-thick membrane (milk fat globule membrane, MFGM) derived from the apical cell membrane of the Golgi apparatus and from membrane parts of the lactating cell added before exocytosis; the MFGM comprises layers of proteins, enzymes, and phospholipids (PLs) which constitute an essential part of this membrane (Tetra Pak, 2003; Singh and Gallier, 2017; Figure 1). Milk proteins are represented by soluble whey proteins and caseins (the dominant class), which exist as colloidal particles, casein micelles, in milk plasma (Figure 1); all caseins are phosphoproteins and the phosphate groups also bind large amounts of calcium (Singh and Bennett, 2002).

Irrespective of the environment or farm type, raw milk, due to its rich composition, remains a highly perishable medium that largely determines the quality and safety of fresh and processed dairy products. Milk produced in a healthy udder is believed to be virtually sterile. However, after withdrawal, depending on the production and environmental conditions, various contamination sources (water, silage, milking equipment, ambient air of the farm environment, etc.) promote the entrance of different types of microorganisms (viruses, bacteria, bacterial spores, and fungi) into raw milk. Given their roles as either beneficial or detrimental to humans, among all microbes, bacteria are of major importance. To preserve the quality and safety of raw milk, two options are recommended: in low-income countries, the activation of the lactoperoxidase system (LPS) is used to prevent excessive bacterial growth (FAO, 2005). In high-income countries, where efficient cold-chain conditions exist, raw milk is chilled immediately after milking and kept at low temperatures until processing stages (European Parliament Directive, 2013).

## Persisting Popularity of Raw Milk Consumption

For the period 1993–2012, a total of 127 outbreaks, which were reported to the Centers for Disease Control and Prevention (CDC, United States) and lead to 1909 illnesses and 144 hospitalizations, were linked to the consumption of raw milk or raw milk-based products (ice cream, soft cheese, or yogurt). The belief that raw milk is healthier than pasteurized milk remains strong, and selling raw milk is legal in 30 states, though outbreaks are on the rise in the United States (Centers for Disease Control and Prevention, 2017). Despite the fact that reports regularly point to health risks linked to the consumption of raw milk or of dairy products made from raw milk, especially for vulnerable populations (children, older adults, pregnant woman, and people with a weakened immune system), a trend toward consumption of raw food with the “raw food diet” or “minimally processed food” products has tended to gain in popularity. Consumers neglect that dairy farms also constitute an important reservoir of food-borne pathogens (Verraes et al., 2015; Van Asselt et al., 2017).

In Europe, microbiological risks are more frequently encountered in dairy products than chemical or physical hazards: the public health risks related to drinking raw milk are mostly due to *Campylobacter* spp., *Salmonella* spp., *Escherichia coli* strains producing shigatoxin, *Brucella melitensis*, *Mycobacterium bovis*, and tick-borne encephalitis virus (EFSA, 2015). For the years 2009 to 2014, the Rapid Alert System Food and Feed notifications associated with food safety hazards in dairy products for the most part reported microbiological contamination: 76% of the cases incriminated pathogens (mainly *Listeria monocytogenes*, *E. coli*, and *Salmonella*), whereas 24% were related to spoilage microorganisms (Van Asselt et al., 2017).

## Characteristics of Raw Milk With a Bacterial Load of 3⋅10^5^ cfu/ml at the Processing Stage

### Cold Storage-Triggered Changes

For Europe, the recommendations stipulate that shortly after withdrawal, raw milk should be rapidly chilled and stored in farm tanks at temperatures below 8°C or at not more than 6°C in the case of daily versus not daily (typically every 2nd day) collection, respectively, so that bacterial counts do not exceed 10^5^ cfu/ml; subsequently, the cold-stored milk is transferred into an insulated tank of a truck before being routed, at a temperature that must not exceed 10°C, to dairy silos; before processing at dairies, raw milk is further cold-stored so that the “total bacterial counts” do not exceed 3 × 10^5^ cfu/ml (this legal “total bacterial counts” label corresponds to aerobic vegetative mesophilic types), as counts above this level are associated with defects in dairy products (Sorhaug and Stepaniak, 1997; European Parliament Directive, 2013).

To prevent the separation of skim milk and cream fractions during cold storage, raw milk is constantly subjected to agitation in tanks and silos; the concentrations of dissolved oxygen (O_2_) and nitrogen (N_2_) reach 0.6 and 0.16 mg/100 *g*, respectively, in cold-stored bovine raw milk, whereas in the udder, the dissolved O_2_ level is approximately 0.15 mg/100 *g* (Tetra Pak, 2003; Walstra et al., 2006).

If the storage temperature is a critical factor, as illustrated by the growth inhibition of bacteria at temperatures of 4–5°C for a limited time period, the “time” factor is also of crucial importance, as slightly above 48 h, these low temperatures are not sufficient to prevent the growth of cold-tolerant psychrotrophic bacteria (able to grow below 7°C), of which the vast majority associated with food spoilage at refrigeration temperatures is constituted by aerobes or facultative anaerobes (Chambers, 2002; Tetra Pak, 2003; Jay et al., 2006). Dairy intensification, which has also entailed the replacement of many small local dairies by fewer but larger and more distant dairy industries, has increased the importance of the “time” factor. A recent report indicated that raw milk can be kept in refrigerated conditions for 3 to 5 days in a farm bulk tank before being delivered to dairy processing plants (Vithanage et al., 2016).

The dominant view is that, after withdrawal, bacterial genera vulnerable to low temperatures are outnumbered by others favored by cold-storage conditions: accordingly, gram-positive micrococci and streptococci are supplanted by gram-negative rods such as *Pseudomonas*, *Acinetobacter*, and *Aeromonas*; several studies have indicated that bacterial diversity decreases during cold storage; high-throughput sequencing has also revealed the presence of anaerobic taxonomic groups such as *Bacteroides*, for example, which are typically associated with gut microbiota (rather indicative of fecal contamination); moreover, even short-term storage periods lead to quick population changes (Chambers, 2002; Dogan and Boor, 2003; Raats et al., 2011; Quigley et al., 2013; Gschwendtner et al., 2016; Kable et al., 2016).

At the end of cold storage, raw milk undergoes various heat treatments (including pasteurization, UHT, etc.) which aim to eradicate pathogens and increase the shelf life of dairy products. Although most if not all contaminating bacteria (including psychrotrophs) will be destroyed, the same heat treatments activate various bacterial spores present in raw milk, which can germinate even at low temperature; these types are represented by *Bacillus cereus*, a common milk contaminant responsible for foodborne illness, or *Bacillus* spp. and *Paenibacillus*, the key microorganisms limiting the shelf life of fluid milks, whereas *Clostridium tyrobutyricum* is associated with spoilage of long-ripened cheeses (Tetra Pak, 2003; Stenfors Arnesen et al., 2008; Mato-Rodriguez and Alatossava, 2010; Ivy et al., 2012).

### Bacterial Metabolism, Spoilage Features, and Antibiotic Resistance

During cold storage, various primary and secondary bacterial metabolites are released in raw milk. For example, the presence of toxins (Verraes et al., 2015; Mylius et al., 2018), cyclic lipodepsipeptides (CLPs; Reybroeck et al., 2014), lysophospholipids (Keenan and Mather, 2002; Gallier et al., 2010; Munsch-Alatossava et al., 2018a), and autoinducers (Christensen et al., 2003; Lu et al., 2004) has been evidenced. If most bacterial cells and heat-labile compounds present in raw milk are inactivated by subsequent heat treatments, the risk that heat-stable metabolites may be displaced into dairy products cannot be excluded.

**FIGURE 2:**
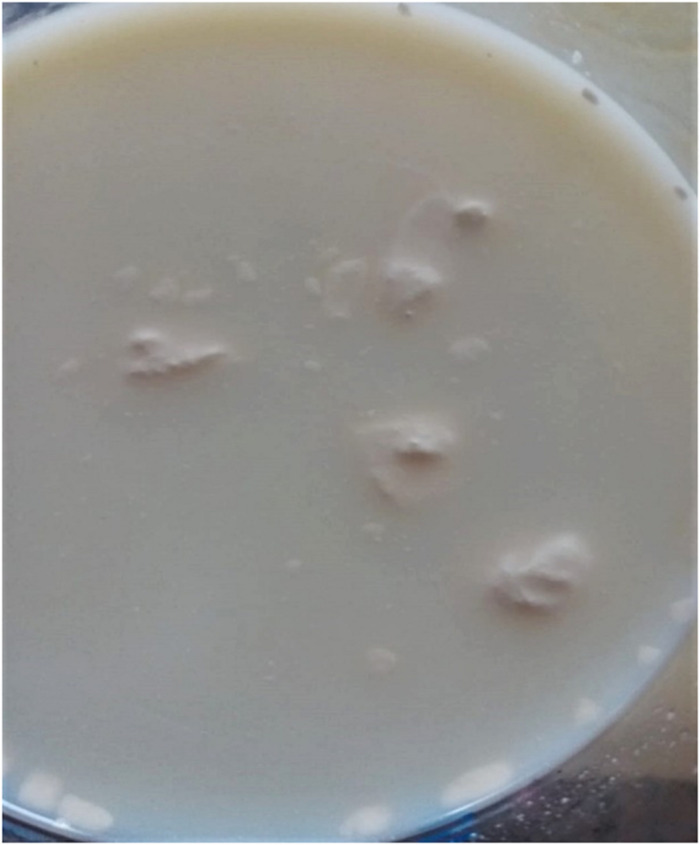
Spoilage assessment by the consumer. The UHT milk (labeled best before 30.03.20) was purchased on 29.12.19 and opened on 30.12.19. The six bottles, belonging to the same pack, appeared perfectly sealed, and all presented the same defect: the numerous aggregates of variable sizes most likely result from proteolytic activity on casein micelles. Excessive thermoresistant protease activities may be due to raw milk-associated psychrotrophs or to deficiencies that occurred at any stage of the milk-processing chain, including the presence of BFs.

Normal bovine milk contains tens of indigenous enzymes: many are technologically significant as indicators of deterioration, thermal history, or mastitis infection (Fox, 2003). However, most enzymes associated with spoilage and «technological» complications, or failures are of bacterial origin. Psychrotrophic bacteria that thrive in cold-stored raw milk produce and excrete various spoilage enzymes such as proteases, lipases, and phospholipases (PLases), which target the different milk components; unfortunately, many of these enzymes show remarkable inherent heat stability as they withstand pasteurization or even UHT treatments (Cousin, 1982; Shah, 1994; Sorhaug and Stepaniak, 1997; Chambers, 2002; Dogan and Boor, 2003); one recent report indicated that over 30% of the investigated *Pseudomonas* strains retained residual lipase and protease activities after being subjected to 142°C for 4 s (Vithanage et al., 2016). These remaining enzymatic activities can subsequently hydrolyze original milk components, leading to bio/chemical, and physical changes in dairy products; excessive protease activities are associated with bitter flavor and gelation of fluid milk (Figure 2), whereas high levels of lipase promote the apparition of off-flavors or even hinder cheese production; additionally, PLases, by degrading the MFGM, will offer free access to native or bacterial lipases, promoting the hydrolysis of neutral lipids normally “stored” inside the fat globules (Chrisope and Marshall, 1976; Cousin, 1982; Deeth, 2002; Figure 1). Ultimately, in raw milk, lipolysis results in a drop in the pH and an increase in the content of free fatty acids (FFAs; Deeth, 2002; Tetra Pak, 2003). Altogether, an excess of bacterial enzymes reduces the shelf life and decreases the quality of dairy products. Combinations of higher temperatures for increased times would certainly lead to higher inactivation rates of these enzymes; however, such treatments would simultaneously promote the undesired denaturation of whey proteins (crucial, for example, for cheese manufacture), and the destruction of vitamins, with notable impact on both organoleptic and nutritional features of milk.

Food has also been identified as one main direct vehicle for the transmission of antibiotic-resistant bacteria from animals to humans and of antibiotic resistance (AR)-encoding genes carried by zoonotic bacteria (Sørum and L’Abbé Lund, 2002; Wichmann et al., 2014). Similarly, raw milk-associated psychrotrophs and mesophiles, which frequently exhibit spoilage features, also carry AR features; strangely enough, AR was most prevalent in both population types at a stage when the total bacterial counts reached approximately 10^5^ cfu/ml; notably, the raw milk samples presented simultaneously significant amounts of multiresistant bacteria (Munsch-Alatossava and Alatossava, 2007; Munsch-Alatossava et al., 2012, 2017).

## Quorum Sensing Also Regulates the Expression of Bacterial Spoilage Enzymes in Raw Milk

For gram-positive or gram-negative bacterial types, the cell density-dependent signaling system, or quorum sensing (QS) is involved in major functions/activities such as sporulation, competence, competition, the production of toxin, exopolymers, and extracellular DNA (eDNA), and virulence gene expression. QS also promotes biofilm formation, including the production of enzymes (Davies et al., 1998; Miller and Bassler, 2001; Papenfort and Bassler, 2016). The signaling compounds or autoinducers are of various types: gram-negative bacteria synthesize fatty-acid derivatives, N-acyl-homoserine lactones (AHLs) also called AI-1, which mostly serve in intraspecies communication; both gram-positive and gram-negative bacteria produce AI-2, a furanosyl borate diester or furanone derivative, considered the universal signaling molecule, that enables interspecies and intraspecies communication (Miller and Bassler, 2001; Smith et al., 2004; Papenfort and Bassler, 2016).

Signaling compounds have also been isolated from foods (Smith et al., 2004; Skandamis and Nychas, 2012), including milk and dairy products: experiments have established that several hydrolytic enzymes produced by *Serratia proteamaculans* and involved in milk spoilage were regulated by QS (Christensen et al., 2003). Additionally, AHL production is a common trait of psychrotrophic bacteria present in cooled raw milk, and AI-2 activity is produced by bacteria associated with the smear of surface-ripened cheeses (Pinto et al., 2007; Gori et al., 2011). Interestingly, AI-2 activity was detected in regular and organic milk even though the bacterial populations were lower than 2 log cfu/ml, but pasteurization and ultra-pasteurization did not completely neutralize the activity, corroborating earlier observations about AI-2 activity, which proved to be acid stable, base labile, and resistant until 80°C (with a loss of activity at 100°C; Surette and Bassler, 1998; Lu et al., 2004). Moreover, the analyses of cell-free samples revealed the presence of AHLs in raw milk as well as in pasteurized and UHT-treated milk (Ammor et al., 2008).

## Biofilms in Dairies

### Generalities

Considering the high availability of nutrients, biofilms (BFs) are very widespread in both the food and beverage industries. In dairies, aside from some rare exceptions (Lortal et al., 2009; Bassi et al., 2017), BFs are mostly associated with continuous hygiene problems and serious economic losses (Carpentier and Cerf, 1993; Sharma and Anand, 2002; Marchand et al., 2012). Surface contamination of food processing lines plays a major role in food contamination; therefore, strict regulations regarding materials in contact with food are applied. Similarly, storage, transportation, and processing of raw milk from farms to dairies occur in storage and process tanks and tubes through which milk circulates, which are mostly made of stainless steel, widely favored for its neutrality, easiness to clean, thermal conductivity, and resistance to harsh detergents; however, some smaller pieces such as seals and gaskets are made of rubber (Tetra Pak, 2003). The rich composition of milk directly provides components that contribute to BF development (Figure 1); bacterial activities furnish other crucial compounds such as exopolysaccharides (EPS), teichoic acids, and extracellular DNA (eDNA; Gross et al., 2001; Sutherland, 2001; Schwartz et al., 2016).

Most if not all gram-positive bacteria, including spore formers, and gram-negative bacteria present in raw milk have the ability to form BFs; both spores and vegetative cells can attach to stainless steel and fouled surfaces; in addition, sporulation occurs within BFs (Wijman et al., 2007; Marchand et al., 2012; Faille et al., 2014).

Consequently, multi-generic or multispecies BFs can be found in dairies (Marchand et al., 2012). In dairy manufacturing plants, two types of BFs are distinguished: environmental BFs, which form in the processing environment (in places difficult to clean) and process BFs, which are established on process line surfaces in contact with the flowing product or on ultrafiltration and reverse osmosis membranes, as well as on vulnerable parts such as T-junctions or “dead legs” prone to fouling; process line BFs are characterized by rapid growth (within hours) whereas environmental BFs usually grow slower; mostly, in BFs associated with process equipment, one or a few species dominate, in contrast to environmental BFs, which are characterized by higher bacterial diversity (Carpentier and Cerf, 1993; Chambers, 2002; Bremer et al., 2015).

### Some Physicochemical Factors That Affect Biofilms in Dairies

The diversity and remarkable adaptability features of bacteria and spores present in raw milk allow them to thrive at any temperature in dairies; this point is illustrated by the ability of these organisms to grow at even higher temperatures in BFs than the usual growth temperature for the planktonic state (Teh et al., 2012). Unsurprisingly, some particular traits of psychrotrophs such as the capacity to produce higher yields of enzymes and polysaccharides at low temperatures allow psychrotrophic bacteria to dominate in BFs established during cold storage (Jay et al., 2006). On the other hand, the obligate thermophilic spore formers such as *Geobacillus* sp. and *Anoxybacillus* sp., most prevalent in dairy powders, will become established on processing lines where temperatures between 40 and 65°C support their growth as well as BF formation; the simultaneous mesophilic and thermophilic features of *Bacillus licheniformis*, another spore former also commonly isolated in dairy powders, make it a major dairy continuum contaminant (Zhao et al., 2013; Flint et al., 2015; Kent et al., 2016). Their persistence in BFs is largely due to the overcoming of the selective pressures in the dairy manufacturing plant, as these bacterial types have perfectly adapted to heat, pH, water availability, and specific product composition (Burgess et al., 2010).

The pH values from fresh unspoiled raw milk range from 6.6 to 6.8 at 20°C, but prolonged cold storage promotes a decrease in raw milk pH; several reports have shown that lower pH values favor BF formation (Skura et al., 1986; Dechemi et al., 2005; Dat et al., 2012; Atulya et al., 2014; Miranda et al., 2018; Munsch-Alatossava et al., 2019).

Milk is continuously exposed to air (with an oxygen content of 21%) at the milking, handling, and transportation stages from farms to dairies. The presence of O_2_ is associated with two drawbacks: the problem of maintaining the quality of milk as the auto-oxidation process deteriorates the flavors and nutritional properties of raw milk and dairy products (Walstra et al., 2006) and the resulting bacterial development as psychrotrophs are also favored by aeration (Jay et al., 2006). The impact of aeration on the generation time of *Pseudomonas fluorescens*, a common inhabitant of raw milk, when examined within a temperature range of 4 to 32°C revealed that the greatest effect of aeration occurred at 4 and 10°C (Olsen and Jezeski, 1963). As shown for other BF types, bacteria cope remarkably well with variable oxygen/air concentrations within dairy BFs: as observed for *P. fluorescens* (Spiers et al., 2003) or *B. cereus* (Wijman et al., 2007), the thermophilic spore formers *Anoxybacillus* and *Geobacillus* also preferentially established BFs at air–liquid interfaces rather than on submerged surfaces (Zhao et al., 2013).

In the dairy, as for other environments, the physicochemical features of the material surface greatly determine the efficiency of bacterial attachment and BF formation. Several reports indicate that the charge and hydrophobicity of the surface constitute the major components that influence bacteria–surface interactions; moreover, the surface roughness or its topographic configuration may also contribute to bacterial attachment and BF formation (Palmer et al., 2015; Berne et al., 2018; Achinas et al., 2019).

### Native Milk Components That Contribute to Biofilm Establishment

The influence of individual milk components on BFs was highlighted by the observations that lactose and non-casein protein solutions yielded the greatest numbers of cells attached on surfaces, whereas skim milk reduced the attachment of *Staphylococcus aureus*, *Serratia marcescens*, and *L. monocytogenes* on stainless steel; moreover, BF formation by *B. cereus* isolates was impaired by a ten-fold dilution of whole milk, and α- and β*-*casein*s* were described as the primary inducers of BF growth for *Streptococcus uberis* (Speers and Gilmour, 1985; Barnes et al., 1999; Shaheen et al., 2010; Varhimo et al., 2011). Sequential changes that lead to conditioning of the stainless steel surface, allowing the formation of a multilayer, include the adsorption of β-lactoglobulin (the major whey protein) at neutral pH on the negatively charged surface; in the following, κ-casein protein adsorbs on the previous layer (this step is favored by a temperature increase; Kim and Lund, 2007; Bassi et al., 2017).

Lactose, the major carbohydrate in milk, has also been reported to trigger BF formation by *Streptococcus mutans* and induce the formation of BF bundles and colony-type BFs for *Bacillus subtilis* (Assaf et al., 2015; Duanis-Assaf et al., 2016).

Divalent cations can influence BF formation directly by reinforcing the cohesiveness of the structure when Ca^2+^ cations bond polymer molecules (Turakhia and Characklis, 1989; Carpentier and Cerf, 1993) or indirectly when Ca^2+^ and Mg^2+^ affect bacterial attachment (Flint et al., 2015).

### Biofilm Formation Favored by the Degradation of Native Milk Components and by the Presence of Secondary Metabolites

Importantly, BF formation in dairies is also promoted by different products resulting from the different hydrolysis reactions that target various milk components. For example, the extracellular proteolytic activity of *S. uberis* is suggested to contribute to increased BF formation (Varhimo et al., 2011). However, regarding proteinases/proteases, bacterial-associated proteolytic activities impact BFs differently. Various lactic acid bacteria produce cell wall-bound proteinases; for lactococcal cells, the proteinase PrtP exhibits a double function, as in addition to its role in milk casein degradation it confers bacterial cells greater hydrophobicity and adhesion to solid surfaces (El Soda et al., 1986; Habimana et al., 2007). A recent report highlighted a similar role for the proteinase PrtS that mediates BF formation by one *Streptococcus* strain (Bassi et al., 2017). However, extracellular protease activity is also required for *Staphylococcus aureus* BF dispersal (Boles and Horswill, 2008). Interestingly, two functions were also assigned to RbmA, a matrix protein that surrounds *Vibrio cholerae* cells; apart from contributing to a reinforcement of the BF structure, the *in situ* limited proteolysis of RbmA promoted the recruitment of planktonic cells (which had not initiated VPS polysaccharide synthesis at the time; Smith et al., 2015).

Interestingly, one study showed that butyric acid, released during lipolysis or milk fat hydrolysis, induced the formation of BF bundles in several *Bacillus* species (such as *B. cereus, B. subtilis* and *B. licheniformis*; Pasvolsky et al., 2014).

Several studies reported that bacteria alter their membrane-lipid composition when entering a biofilm lifestyle by incorporating free exogenous FAs from the host or from the environment (Giles et al., 2011; Hobby et al., 2019); additionally, *Pseudomonas aeruginosa* responds to exogenous polyunsaturated fatty acids (PUFAs) by modifying its phospholipid membrane composition and phenotypes associated with virulence including BF formation, which was enhanced by several PUFAs (Baker et al., 2018).

*Pseudomonas aeruginosa*, also a causative agent of bovine mastitis, has been isolated from raw milk (Dogan and Boor, 2003; Bannerman et al., 2005; Munsch-Alatossava and Alatossava, 2006). Both in raw milk or in post-pasteurization contamination situations, bacterial phospho/lipolytic activities at planktonic or sessile stages provide fatty acids including oleic acid, the principal unsaturated fatty acid in milk (Figure 1). Interestingly, it was shown that *P. aeruginosa* contains a fatty-acid diol synthase that catalyzes the stereospecific oxygenation of exogenous oleic acid; the production and sensing of oleic acid-derived “oxylipins” regulate BF formation, and oxylipins are at the basis of a novel quorum sensing system (ODS) believed to be more widely distributed among bacterial species (Martinez and Campos-Gómez, 2016; Martinez et al., 2019). Notably, a recent study showed that oxylipins detected in bovine milk were mainly composed of linoleic (C18:2) and α-linolenic acid (C18:3) metabolites (Goncalves Dias et al., 2020).

Phospholipolysis, caused by various bacterial phospholipases, promoted an increase in the amount of lysophospholipids (LPE, LPC, LPI, and LPS) in raw milk during cold storage (Munsch-Alatossava et al., 2018a). A link between lysophospholipids and BFs was established for *P. aeruginosa*, as host-derived LPC (lysoPC) modulated the expression of protein RahU involved in the regulation of BF formation (Rao et al., 2011); for *Salmonella*, it was shown that the production and secretion of active flagellin (the principal component of flagella) occurred in response to the sensing of host-produced lysophospholipids (Subramanian and Qadri, 2006). Flagella exert a critical role in lifestyle switch during BFs attachment and detachment stages; in the later, *P. aeruginosa* cells synthesized again flagellin before returning to the planktonic state (Suriyanarayanan et al., 2016; Berne et al., 2018).

Both *Bacillus* and *Pseudomonas* species, well represented in raw milk, are well known for producing secondary metabolites such as CLPs. Among their various biological roles, CLPs are involved in bacterial surface attachment and BF formation; the type of CLP determines whether the producing bacteria are supported in BF formation such as massetolide A or xantholysin, or whether the CLPs inhibit BF formation (Kuiper et al., 2004; De Bruijn et al., 2008; Rokni-Zadeh et al., 2012; Li et al., 2013). Interestingly, the presence of CLPs was evidenced in raw milk and found to “falsify” antibiotic residue detection (Reybroeck et al., 2014).

### Repercussions of Dairy Biofilms

The consequences of dairy-associated BFs are multifaceted: compared to planktonic cells, sessile bacteria show an enhanced production of proteases and lipases (Teh et al., 2012, 2013). BF dispersal inevitably leads to contamination of raw, heat-treated milk, and dairy products, as many enzymes produced at BF stages also show remarkable heat stability and their activities promote off-flavors in end products. The presence of BFs on dairy equipment is also associated with an increased corrosion rate, reduced heat transfer, and increased fluid frictional resistance (Kumar and Anand, 1998; Bremer et al., 2006).

Bovine mastitis, the inflammatory reaction of udder tissue in response to microbial infection, is very difficult to control in dairy farms; the disease has a very high incidence in these farms and causes significant economic losses worldwide (FAO, 2014; Rollin et al., 2015; OECD-FAO, 2019). A significant number of bacterial types can cause mastitis while simultaneously having the ability to form BFs inside the udder (Schönborn and Krömker, 2016). Among the mastitis-associated bacteria (*Staphylococcus aureus, Streptococcus* spp., *E. coli*, *Salmonella* spp., etc.), some are also human pathogens and can be transmitted via milk (Van Asselt et al., 2017), especially if no preliminary heat treatment takes place.

Particular recalcitrant-thermophilic spore former-based BFs necessitate costly precautions such as intensive product controls, frequent cleaning, and shorter production runs (Zhao et al., 2013). Moreover, the use of various detergents and disinfectants, together with insufficient rinsing operations, is associated with the presence of existing and emerging residues in milk and dairy products; for example, residues of iodine and quaternary ammonium compounds have been found in milk, and an excessive level of hydrogen peroxide was recorded in butter (Danaher and Jordan, 2013; Van Asselt et al., 2017).

## Anti-Biofilm Strategies in Dairies

In the food industry, the control of the quality and safety of food relies on prevention means such as the strict application of Good Manufacturing Practices (GMP) and Hazard Analysis of Critical Control Points (HACCP; Jay et al., 2006). In dairies, the cleaning (elimination of soiling), and disinfection (destruction of microorganisms) operations of food manufacturing materials typically occur through the “Cleaning-in-Place” (CIP) procedure, which is based on high-velocity passage of hot water and alkaline/acidic detergents, followed by hot water rinsing of the equipment surfaces. Deficiencies in sanitation programs or limitations of CIP regimes (due to the high resistance of spores) inevitably lead to BF formation and extension in dairy plants (Carpentier and Cerf, 1993; Austin and Bergeron, 1995; Sharma and Anand, 2002; Tetra Pak, 2003, Bremer et al., 2006; Faille et al., 2014).

The magnitude of the problems caused by BFs is reflected by the diversity of the investigated anti-BF strategies including the control of milk pH (Dat et al., 2012), the application of a modified CIP regime (with the addition of enzymes, for example; Paul et al., 2014); the use of bacteriophages, phage lysins (Gutierrez et al., 2016), and bacteriocins (with individual or combined use; Cleveland et al., 2001; de Pimentel-Filho et al., 2014); the coating of surfaces with peptides (Friedlander et al., 2019); the application of ultrasonication (Oulahal et al., 2004); the use of ozone (Khadre and Yousef, 2001; Varga and Szigeti, 2016); and the enrichment of milk with at least 5 mM magnesium (Ben-Ishay et al., 2017); however, no effective technological solution exists yet (Friedlander et al., 2019).

## N_2_ Gas Flushing Technology

### Major Results With Raw Milk

To prevent bacterial spoilage of raw milk during cold storage, two studies evaluated the use of N_2_ gas applied in a “closed system” (Murray et al., 1983; Dechemi et al., 2005). By considering an “open system” somehow more realistic considering dairy equipment, a pure N_2_ gas flushing-based technology was devised and tested: briefly, sterile-filtered N_2_ gas is continuously flushed through the headspace of a milk-containing vessel (Munsch-Alatossava et al., 2010c,b; Alatossava and Munsch-Alatossava, 2018). The method proved to be of interest as an additional or alternative treatment by hindering bacterial development and simultaneously preventing the bio/chemical degradation of raw milk components during cold storage (Table 1).

**TABLE 1:** Summary of the advantages determined with the use of N_2_ gas.

**Microbiological aspects**	**Effects**	**References**
	Inhibition of bacterial growth at low and milder temperatures at laboratory scale (culture-based studies)	Murray et al., 1983; Skura et al., 1986; Dechemi et al., 2005; Munsch-Alatossava et al., 2010c, 2016, 2018a
	“Exclusion” of phospholipase producers and vegetative cells of the *Bacillus cereus* type (culture-based studies) in raw milk	Munsch-Alatossava et al., 2010c,a
	Inhibition of bacterial growth at pilot plant scale (100 L)	Munsch-Alatossava et al., 2010b
	Better preservation of the initial bacterial diversity during raw milk cold storage; Inhibition of *Pseudomonas* and *Acinetobacter* (next-generation sequencing-rRNA-based study)	Gschwendtner et al., 2016
	Bactericidal against *Bacillus weihenstephanensis*	Munsch-Alatossava and Alatossava, 2014
	Anaerobes are not favored	Munsch-Alatossava et al., 2010c; Gschwendtner et al., 2016
	Similar efficiency as that of the activated lactoperoxidase system at milder temperatures	Munsch-Alatossava et al., 2016
	Dissemination of antibiotic resistance is hindered	Munsch-Alatossava et al., 2017
	Sporulation is not promoted	Munsch-Alatossava et al., 2013; Munsch-Alatossava and Alatossava, 2014

**Biochemical aspects**	**Prevents or alleviates**	**References**

	Proteolysis	Murray et al., 1983; Skura et al., 1986; Dechemi et al., 2005; Munsch-Alatossava et al., 2019
	Lipolysis	Murray et al., 1983; Dechemi et al., 2005; Munsch-Alatossava et al., 2019
	Phospholipolysis	Munsch-Alatossava et al., 2018a
	Auto-oxidation	Gursoy et al., 2017; Munsch-Alatossava et al., 2019
	Maintains initial raw milk pH	Murray et al., 1983; Dechemi et al., 2005; Munsch-Alatossava et al., 2010c, 2019

### Studies With Gram-Positive and Gram-Negative Bacterial Representatives

**FIGURE 3:**
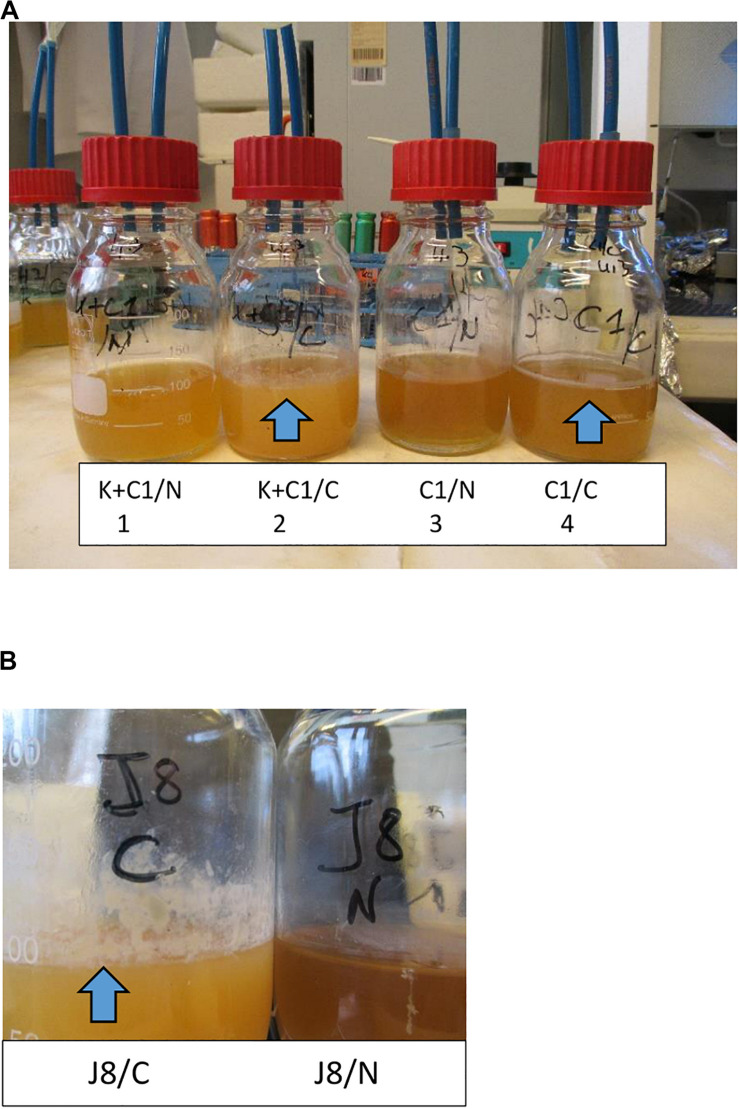
N_2_ gas flushing inhibits BF formation. The presence of BFs is depicted by arrows. **(A)** Bacterial cultures grown in BHI broth for 6 days at 25°C. The conditions were as follows: (1) co-culture of KBAB4 (K) and C1, N_2_-flushed; (2) co-culture of KBAB4 (K) and C1, as a control (ambient air); (3) monoculture of C1, N_2_-flushed; and (4) monoculture of C1, as a control. The bacterial counts were detailed in a previous study (Munsch-Alatossava and Alatossava, 2014). **(B)** The raw milk isolate J8 was grown in BHI broth for 10 days at 6°C. J8/C accounts for the control (ambient air), whereas J8/N corresponds to the N_2_-flushed culture.

When the N_2_ gas flushing treatment was applied to the gram-negative *P. fluorescens* raw milk isolate C1, its growth was inhibited during the exponential growth phase compared to the control conditions (ambient air) at both considered temperatures (6 and 12°C; Munsch-Alatossava and Alatossava, 2006; Munsch-Alatossava et al., 2010c); investigations with the gram-positive spore former *Bacillus weihenstephanensis* (strain KBAB4) revealed a temperature-dependent behavior: the treatment promoted the emergence of small colony variants (SCVs) at 15°C, but most importantly, scanning and transmission electron microscopy analyses revealed that the treatment triggered cell death at 25°C (Munsch-Alatossava and Alatossava, 2014). Mono- and co-cultures of both C1 and KBAB4 strains in Brain Heart Infusion (BHI) broth showed the presence of BFs (rather diffuse for the co-culture and denser for the monoculture) on the surface of the test bottles used for the cultivation of the controls (C; conditions 2 and 4, Figure 3A), in contrast, to the bottles that received the additional N_2_-flushing treatments (N; conditions 1 and 3), for which no such observations were made (Figure 3A). *Pseudomonas* sp. raw milk isolate J8 (Munsch-Alatossava and Alatossava, 2006), cultured in BHI broth, showed a similar behavior: development of a BF in the control conditions (C; ambient air), whereas no BF was apparent when the N_2_-flushing treatment was applied (N; Figure 3B). Hypotheses on possible modes of action of N_2_ flushing on gram-positive or gram-negative bacterial types were discussed in a previous report (Munsch-Alatossava et al., 2018b).

## How Could N_2_ Gas Flushing Contribute to Preventing Biofilm Formation?

**FIGURE 4:**
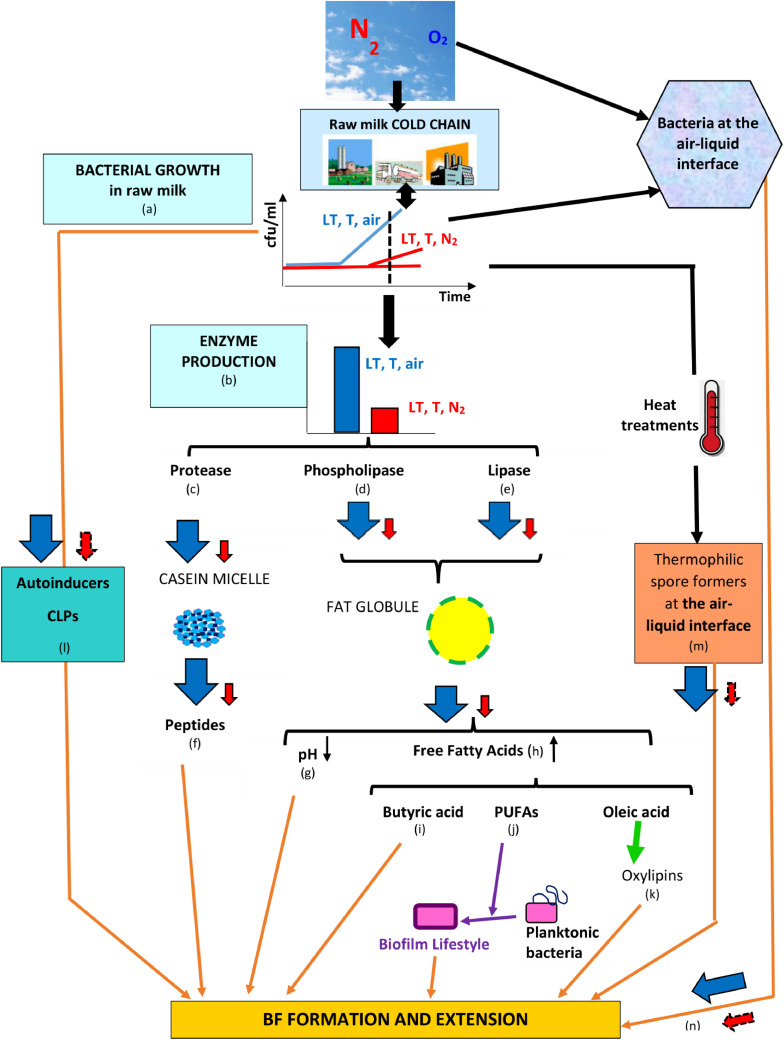
Series of counter-events, triggered by N_2_ flushing, that could contribute to reducing BF formation in dairies. Legend: **LT, T**, and **air** = low temperature for some time at ambient atmosphere (78% N_2_, 21% O_2_); **LT, T**, and **N_2_** = low temperature for some time under N_2_ flushing (∼100% N_2_); the changes induced by LT, T, air and LT, T, N_2_ are represented by 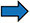 and 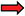, respectively, whereas presumptive changes are symbolized by 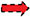. The relevant references are listed as follows: **(a)** Chambers (2002), Raats et al. (2011), Quigley et al. (2013), Gschwendtner et al. (2016), Dechemi et al. (2005), Munsch-Alatossava et al. (2010c); **(b)** Murray et al. (1983), Chambers (2002), Dechemi et al. (2005), Munsch-Alatossava et al. (2010c, 2018a, 2019), Vithanage et al. (2016); **(c)** Murray et al. (1983), Skura et al. (1986), Dechemi et al. (2005), Munsch-Alatossava et al. (2010c, 2019); **(d)** Munsch-Alatossava et al. (2018a); **(e)** Dechemi et al. (2005), Munsch-Alatossava et al. (2010c, 2019); **(f)** El Soda et al. (1986), Habimana et al. (2007), Varhimo et al. (2011), Bassi et al. (2017); **(g)** Skura et al. (1986), Deeth (2002), Dechemi et al. (2005), Walstra et al. (2006), Dat et al. (2012), Atulya et al. (2014), Munsch-Alatossava et al. (2010c, 2019); **(h)** Deeth (2002), Taylor and Mac Gibbon (2002), Walstra et al. (2006), Dechemi et al. (2005), Munsch-Alatossava et al. (2019); **(i)** Pasvolsky et al. (2014); **(j)** Giles et al. (2011), Baker et al. (2018), Hobby et al. (2019); **(k)** Martinez and Campos-Gómez (2016), Martinez et al. (2019); **(l)** Reybroeck et al. (2014), Lu et al. (2004), Ammor et al. (2008); **(m)** Zhao et al. (2013); and **(n)** Wijman et al. (2007), Spiers et al. (2003).

All studies that evaluated N_2_ gas-based treatments in “closed” or “open” systems showed that the treatment overcomes some major drawbacks underlying “cold storage for a certain time,” concluding that aerobic and facultative anaerobic vegetative bacterial development was impeded, anaerobes were not favored, and some bacterial groups associated with spoilage were particularly targeted; additionally, the activities of bacterial lipase, protease, phospholipases C, A, and D, and sphingomyelinase C were reduced and coincided with little or no bacterial growth in cold-stored N_2_-flushed raw milk (Table 1). The fact that the N_2_ treatment better preserves original milk components implies that lower amounts of bacterial primary metabolites are released in raw milk (Figure 4); if the flushing treatment would be applied from farms to dairies, a fair assumption would be that raw milk with lower bacterial loads would enter dairies. Moreover, lower bacterial loads imply that lower amounts of EPS would be available and that less eDNA and lower amounts of secondary metabolites such as toxins, CLPs, and autoinducers would be released in raw milk, which could altogether contribute to hindering BF establishment or extension in dairies (Figure 4); presumably, lower levels of autoinducers in N_2_-flushed raw milk would not benefit eventual post-pasteurization contaminants.

Pure N_2_ gas flushing prohibited sporulation for *B. weihenstephanensis*, irrespective on whether the strain was grown in mono- or co-cultures (Munsch-Alatossava and Alatossava, 2014). Furthermore, total spore levels in raw and pasteurized milk did not increase, compared to their initial levels, with the flushing treatment (Munsch-Alatossava et al., 2013, 2018a). Similar observations with oxygen-limited conditions were reported: for *Bacillus stearothermophilus*, the sparging of cultures with nitrogen instead of air inhibited spore formation (Long and Williams, 1960); similarly, in oxygen-limited conditions, sporulation of one *Bacillus thuringiensis* strain was reduced (Avignone-Rossa et al., 1992).

The heat stability of certain compounds such as autoinducers (for example AI-2; Surette and Bassler, 1998; Lu et al., 2004; Ammor et al., 2008) and CLPs (Reybroeck et al., 2014), present in food materials including dairy products, raises questions about their outcomes and eventual interactions with the gut microbiota. Interestingly, a recent study showed that mammalian epithelial cells produce a mimic of the bacterial autoinducer AI-2 in response to secreted bacterial factors; the authors also suggest that cross-kingdom communication occurs between eukaryotic cells and bacteria via the AI-2 bacterial quorum sensing system (Ismail et al., 2016).

By creating conditions that lower the availability of free oleic acid (Figure 4), N_2_ flushing could also disturb the oxylipin-based QS circuitry and hence contribute to limiting BF formation or extension by bacterial types that rely on ODS (Martinez et al., 2019; Figure 4).

Considering limitations underlying the recommended raw milk preservation methods (FAO, 2005; Munsch-Alatossava and Alatossava, 2007; Munsch-Alatossava et al., 2012; Teh et al., 2012; Botelho et al., 2015) together with problems caused by BFs in dairies (Carpentier and Cerf, 1993; Sharma and Anand, 2002; Marchand et al., 2012), future actions that aim to ensure microbial food safety will inevitably imply considering climate change and more precisely how to maintain the cold chain of raw milk storage and transportation when ambient temperatures are rising. Technological solutions that could prevent or hinder BF formation in dairies would result in less spoilage, fewer outbreaks, and use of less water and detergents, which could ultimately benefit dairy farms, industries, and consumers.

That the N_2_ flushing treatment could exhibit anti-biofilm properties is somewhat directly suggested from the observation of mono- and co-cultures of bacterial strains (Figure 3); however, further studies are necessary to precisely evaluate and quantify anti-BF properties of a pure N_2_-based atmosphere.

## Conclusion

Future actions that ameliorate food waste and losses, while ensuring microbial food safety, must address sustainable development goals established by the United Nations (Sustainable Development Goals, 2015) in a context where higher ambient temperatures will inevitably impose increasing pressure on maintaining the cold chain of food storage and transportation.

Strategies that aim to achieve better control of BFs are divided in either preventing BF formation or targeting mature BFs. In dairies, as elsewhere, the formation of BFs is conditioned by the balance between favorable and antagonistic factors; by reducing the beneficial effects exerted by favorable factors of either a microbiological or biochemical type, N_2_ gas flushing could contribute to preventing or limiting BF formation and extension.

## Author Contributions

PM-A wrote the 1st draft of the manuscript. TA contributed to data compilation related to biochemical and dairy technological aspects and critically read and commented the manuscript. Both authors contributed to the article and approved the submitted version.

## Conflict of Interest

The authors declare that the research was conducted in the absence of any commercial or financial relationships that could be construed as a potential conflict of interest.
